# Notes and News

**Published:** 1888-01-14

**Authors:** 


### NOTES AND NEWS.

COLLECTED AND COMMUNICATED.

DR. J. S. BRISTOWE, F.R.C.P.Lond., F.R.S., Senior Physician to St. Thomas's Hospital, has accepted the Presidency of the Hospitals Association *vice* Sir Andrew Clark, resigned. Sir Edmund Hay Currie, Mr. E. H. Lushington (treasurer of Guy's Hospital), and Mr. F. C. Carr-Gomm (chairman of the London Hospital), have also joined the Council of the Association. The appointment of Dr. Bristowe is one which does honour to the foresight of the Council. Dr. Bristowe is a man of philosophic temper, sober judgment, and quiet firmness of mind, and enjoys in an unusual degree the confidence both of the public and the medical profession.

THE sum of £1,507 has been handed to the authorities of Lincoln Hospital as the result of a bazaar held on behalf of that institution.

WE note with pleasure that the awards for Newcastle International Exhibition have now been made, and that Spratt's Patent (Limited) received a prize medal and the highest award in their class, the jury making especial note of the superior excellence of their ship biscuits.

H.R.H. PRINCESS LOUISE, Marchioness of Lorne, has graciously consented to act as a patroness of the fancy dress ball to be held on the 9th February next at the Hotel Metropole, in aid of the funds of the North London (University College) Hospital.

MISS LOCH ("Sister Darker,") of St. Bartholomew's Hospital, has been appointed matron of the Government Military Hospital at Poona out of several hundred applicants. Miss Oxley, of Guy's Hospital, has also been appointed by the Secretary of State for India, to the charge of the nurses which it is intended to introduce into the military hospitals in India.

WE much regret that in consequence of a misunderstanding as to the date of the paper on the "Amelioration of the Condition of Midwives," read by Dr. Aveling to the members of The Hospitals Association on the 11th January, an abstract of the paper which had been sent to us by the author was published in last week's HOSPITAL, several days before the date of its delivery.

MESSRS. WILSON AND WHITEHOUSE, of Vernon Place, on Saturday last placed an oil painting, executed by them, in the Harper Ward of the Westminster Hospital. The painting has for its subject the figure of St. Luke in the character of a physician—a character in which he is often regarded from the reference to him in Coloss. iv. 14, where St. Paul speaks of him as "The beloved physician." The figure is nearly life-size, and the treatment decorative. Messrs. Wilson and Whitehouse are the artists to whom the "Gordon Memorial" window, in the Manchester Cathedral, has been entrusted

THE Edinburgh Provident Dispensary can show a good record. During the last year 8,000 cases have received medical help through its agency, and the total cost has been £187. Would that other institutions could show as desirable a proportion between means and results.

THE *Salopian and Montgomeryshire Post* announces the death of Miss Minnie Williams, matron of the Cottage Hospital since 1877. In the discharge of her onerous duties, Miss Williams, by her unfailing courtesy and kindness to the patients under her care, won the love and esteem of a wide circle. For the last two years her failing health forced her to delegate more and more of her duties to her sister, Miss Hannah Williams, who possesses many of her sister's qualities. Miss Williams was about thirty-seven years of age. The cause of her death was consumption.

AT the annual meeting of the Northern Counties Hospital for Incurables, held in the Mayor's parlour, Town Hall, Manchester, on the 22nd ult., it was stated that the subscription for the year amounted to £2,065, the donations to £995, and the income from all sources to £5,303. The year closed with a balance due to the bankers of £511. This deficit, the treasurer, Mr. S. L. Helm, said, might be explained partly by the fact that during the year the institution had received no legacies, and partly by the furnishing of the branch hospital at Mauldeth being provided for out of the general fund. The number of patients at present in the hospital is eighty-six.

THE January number of *Work and Leisure*, a little magazine devoted to the interests of women, contains the first of a series of articles entitled "Four Months in a Cottage Hospital." The writer, having been taught by painful personal experience the necessity for "scientific" nursing, takes a course of ambulance lectures, and afterwards goes to assist the matron of a northern hospital. It is her experiences there that she relates. The same journal contains an account of a German magazine, in which a writer suggests that a course of hospital nursing should be made compulsory for women, as military service is for men.

THE following vacancies are announced this week, the amount of salary in each case being given after the name of the institution :—

**Resident Medical Officers.**

Bristol Hospital for Sick Children. House Surgeon.—£100, with rooms and attendance. Doubly Qualified.
City of London Hospital for Diseases of the Chest, Victoria Park, E. Medical Officer.—£100
Radcliff Infirmary, Oxford. House Surgeon —£80.
Royal Surrey County Hospital, Guildford House. Surgeon.—£80.
Stourport Friendly Society's Medical Association. Medical Officer.—£170, with unfurnished residence free from rent and rates, together with paid midwifery and vaccination fees.
Brighton, Hove, and Preston Dispensary. Two House Surgeons. —£140 each, with furnished rooms, coal, gas, and attendance.

**Non-resident Medical Officers.**

Central London Throat and Ear Hospital, Gray's Inn Road. Three Clinical Assistants.—Fees £2 2*s*., for six months.
Royal College of Surgeons of England. Assistant in Pathological Department of the Museum.
Hospital for Sick Children, Great Ormond Street. Clinical Assistant in the Out patient Department.
Hospital for Diseases of the Heart and Paralysis, Soho Square. Anæsthetist.

**Nurses.**

Horton Infirmary, Banbury. Trained Nurse.—£20 to begin with.
Chelsea Infirmary, Cale Street. Nurses, age to exceed 26 years. £18, rising to £24 a year, with board, washing, and uniform, and allowance of £5 10*s*., optional, in lieu of beer. One year's training.
Hospital for Consumption, Brompton. Trained Nurses immediately.
Royal Surrey County Hospital Thoroughly trained Head Nurse for Women's Ward. Age from 26 to 35.—£24 rising to £28, with uniform
West London Hospital, Hammersmith Road A fully-qualified Assistant Nurse, not less than 24 years old.—£24 to commence with.
West Kent General Hospital, Maidstone. Two Lady Nurses, with three years' experience, for Women's Ward and Male Accident Ward. Ages from 25 to 35.—£25, with increase after six months, all found, except uniform.
London and Brighton Association. Several. Apply to Lady Superintendent at Brighton.
Glasgow Maternity Hospital. Several, to train in Midwifery and Monthly Nursing.

---

THE Earl of Derby has generously given £1,000 to St. Mary's Hospital for Women, Manchester.

---

SEVERAL changes are suggested in connexion with the working of the East Suffolk Hospital. The governors desire to do away with the distinctions between honorary surgeons and honorary physicians, and to demand both medical and surgical work from every member of the staff. It is also proposed to reduce the honorary staff to six members, as under the proposed amalgamation the honorary surgeons would have too little to do. At the same time, it is proposed to add an assistant house-surgeon to the resident staff, as the governors feel that in so large a hospital there should always be on the premises a doctor ready to attend to cases of emergency; and it is of course unreasonable to expect that the house-surgeon can spend every minute of his time within the walls of the hospital.

---

AT the half-yearly meeting of the governors of this hospital, Mr. W. Alexander suggested that the executors of persons leaving legacies of ten guineas and upwards should have accorded to them half the privileges of donors of similar sums; that is, they should have a certain number of tickets for the hospital allotted to them during the lifetime of one of their number, to be chosen at the time the legacy was paid. Mr. Alexander pointed out that, owing to legacies and donations from persons now dead, the hospital had an invested capital of £26,000, for which no tickets were granted; and after some discussion, the board agreed to consider his proposition favourably. It will, therefore, be moved by Dr. W. A. Elliston at the next Court, which will be held in January.

HER Majesty the Queen has sent gifts of linen to several of the Metropolitan hospitals.

---

A CONCERT in aid of the Queen Victoria Convalescent Home for Sick Children was given in the Exhibition Hall, Botanic Gardens, Belfast. The large hall was crowded, and the entertainment was a financial as well as an artistic success.

---

IT has been resolved to establish a convalescent fund for women in Worcester, and the committee which has taken up the matter has invited the co-operation of all women in the town and district, feeling that the aid and advice they can give would be of special value in a question concerning their sex.

---

THE "Thomas Knight Hospital at Blyth was opened by the Hon. Lady Ridley on the 21st ult. Mr. Thomas Knight was a shipowner, who lived at Crofton, near Blyth. Always a generous contributor to local charities, it was found after his death that he had left a large sum to found a hospital for the district.

---

WE recently called attention to an excellent proposal which has been made, namely, to provide a floating hospital in the North Sea for the benefit of the fishermen who spend so large a portion of their lives there. The English-speaking portion of the North Sea fishing fleet numbers 12,000; and the fishing industry is one of such importance that it deserves every protection the State and the public can give the brave and hardy men who engage in it. In the storms which the fishing fleet encounters accidents of a more or less serious nature are certain frequently to befal, and the exposure to the weather which fishermen must inevitably undergo is apt to induce many serious diseases. At present, any one who is ill, from whatever cause, must submit to considerable delay—which is proverbially dangerous in all things—before the medical aid of even the nearest port can be obtained; days of unrelieved suffering that may lead to the fatal termination of a disease or injury, which, if seen to earlier, might be easily cured. Under these circumstances the proposed floating hospital is an undeniable desideratum, and it is to be hoped that the necessary funds to start and maintain it will soon be found.

---

IMPORTANT alterations have recently been made in the internal arrangements of the Royal Eye Hospital, St. John Street, Manchester. On the completion of the new buildings in Oxford Road, all the in-patients were removed thither, and the old premises in St. John Street were retained for the use of out-patients only. All serious operations are performed at the Oxford Road Hospital, but during last year no fewer than 10,539 new patients have been seen at St. John Street, the total attendances of these numbering over 50,000, and over 500 operations have been performed there. Since the removal of the in-patients to Oxford Road, where, during the eleven months ending November, 1887, as many as 5,573 new patients have been treated, the St. John Street premises have been greatly improved. The patients' waiting-room has been reconstructed, so as to afford more sitting accommodation; a new consulting-room has been added; and as a considerable number of the patients requiring treatment are infants in arms, the board of management have thoughtfully provided a small room where mothers with their children can sit apart from the other patients, an arrangement which is approved of by all parties. The board of management are fortunate in their medical staff, which consists of Dr. Emrys-Jones, Dr. C. E. Glascott, Dr. Little, and Dr. Mules, with Dr. Hill Griffith as assistant-surgeon.

